# Management of neonatal hyperbilirubinemia: Pediatricians' practices and educational needs

**DOI:** 10.1186/1471-2431-6-6

**Published:** 2006-03-06

**Authors:** Anna Petrova, Rajeev Mehta, Gillian Birchwood, Barbara Ostfeld, Thomas Hegyi

**Affiliations:** 1 Division of Neonatology, Department of Pediatrics, Robert Wood Johnson Medical School-University of Medicine and Dentistry of New Jersey, New Brunswick, New Jersey 08903, USA

## Abstract

**Background:**

Early detection and treatment of neonatal hyperbilirubinemia is important in the prevention of bilirubin-induced encephalopathy. In this study, we evaluated the New Jersey pediatricians' practices and beliefs regarding the management of neonatal hyperbilirubinemia and their compliance with the recommendations made by the American Academy of Pediatrics (AAP) in 1994.

**Methods:**

A survey questionnaire was mailed to a random sample of 800 pediatricians selected from a list of 1623 New Jersey Fellows of the AAP initially in October 2003 and then in February 2004 for the non-respondents. In addition to the physicians' demographic characteristics, the questionnaire addressed various aspects of neonatal hyperbilirubinemia management including the diagnosis, treatment, and follow up as well as the pediatricians' beliefs regarding the significance of risk factors in the development of severe hyperbilirubinemia.

**Results:**

The adjusted response rate of 49.1% (n = 356) was calculated from the 725 eligible respondents. Overall, the practicing pediatricians reported high utilization (77.9%) of the cephalocaudal progression of jaundice and low utilization (16.1%) of transcutaneous bilirubinometry for the quantification of the severity of jaundice. Most of the respondents (87.4%) identified jaundice as an indicator for serum bilirubin (TSB) testing prior to the neonate's discharge from hospital, whereas post-discharge, only 57.7% felt that a TSB was indicated (P < 0.01). If the neonate's age was under 72 hours, less than one-third of the respondents reported initiation of phototherapy at TSB levels lower than the treatment parameters recommended by the AAP in 1994, whereas if the infant was more than 72 hours old, almost 60% were initiating phototherapy at TSB lower than the 1994 AAP guidelines. Most respondents did not regard neonatal jaundice noted after discharge and gestational ages 37–38 weeks as being significant in the development of severe hyperbilirubinemia. However, the majority did recognize the importance of jaundice presenting within the first 24 hours and Rh/ABO incompatibility.

**Conclusion:**

The pediatricians' practices regarding the low utilization of laboratory diagnosis for the quantification of jaundice after discharge and underestimation of risk factors that contribute to the development of severe hyperbilirubinemia are associated with initiation of phototherapy at lower than AAP recommended treatment parameters and recognition of neonatal hyperbilirubinemia as an important public health concern.

## Background

Management of hyperbilirubinemia remains a challenge for neonatal medicine because of the risk for serious neurological complications related to the toxicity of bilirubin [1]. The neonatal hyperbilirubinemia practice guidelines published in 2004 by the American Academy of Pediatrics (AAP) expresses the pediatric community's concern regarding bilirubin-induced neurological pathology [2].

The prevention of bilirubin encephalopathy is based on the detection of infants at risk for developing significant hyperbilirubinemia and the early treatment of this condition [3]. Newman and Maisels [4] have questioned the compliance with the existing guidelines in the neonatal hyperbilirubinemia cases associated with an adverse outcome. Therefore, understanding the pediatricians' practices and beliefs towards the management of neonatal hyperbilirubinemia is of particular importance. A survey conducted more than ten years ago showed wide variation in neonatal hyperbilirubinemia management practices among the pediatricians and neonatologists [5]. Approximately 66% of the pediatricians reported an awareness of the neonatal hyperbilirubinemia clinical practice guidelines published in 1994 [6]. Atkinson et al [7] showed that only 54% of the pediatricians initiated treatment in accord with the recommended parameters [7]. However, none of the previous studies investigated the pediatricians' preferences regarding management of hyperbilirubinemia in term infants before and after hospital discharge. Moreover, no study has clearly assessed the pediatricians' beliefs regarding the risk factors for severe neonatal hyperbilirubinemia.

In the present survey study we evaluated the New Jersey pediatricians' practices and beliefs regarding management of neonatal hyperbilirubinemia and their compliance with the 1994 AAP recommendations.

## Methods

We designed a mailing survey study. The survey questionnaire was mailed to a random sample of 800 pediatricians selected from a list of 1623 New Jersey Fellows of the AAP. The list obtained from the AAP did not specify the physicians' area of practice. The questionnaire was mailed twice to the pediatricians, initially in 2003 (October 27–29) and then in 2004 (February 24–28) for the non-respondents. A letter that assured the participants of the voluntary nature of the study, complete anonymity, and confidentiality of data accompanied the questionnaire.

All respondents were classified as those: (i) who completed more than 80% of questions; (ii) who completed 50% to 80% of questions; (iii) who did not return survey; and (iv) others (questionnaire returned by postal services for the non-availability of a forwarding address). The categories that were not eligible for inclusion in the analysis included: residents in training; retired pediatricians; pediatricians who did not provide services for newborn infants; and pediatricians who answered less than 50% of the questions. The technique of the Council of American Survey Research Organization (CASRO) was used to classify the survey respondents and calculate the response rate [8].

### Study Instrument

We designed a two-page (four-sided) survey questionnaire that included 25 questions. The questions addressed various aspects of neonatal hyperbilirubinemia management such as pre-discharge bilirubin testing and follow up of infants who were jaundiced at discharge, the diagnostic and treatment approaches used for the management of neonatal hyperbilirubinemia, and the public health significance of these conditions. The physicians were also asked about their practice type, the population area covered by their service, years in practice since completing residency, annualized number of neonates seen in their practice, and experience with cases of kernicterus.

All questions were designed using a yes/no and either single or multiple-choice format. A scale type format (hardly at all, to a small degree, to a moderate degree, to a very high degree, and not applicable) was used to assess the pediatrician's beliefs regarding the risk factors for severe hyperbilirubinemia. We assessed the following risk factors: jaundice presenting in first 24 hours, jaundice noted at discharge, previous siblings with jaundice, gestational age between 37 and 38 weeks, breast feeding, bruising/cephalohematoma, Rh and ABO incapability, and glucose-6-phosphate dehydrogenase (G-6-PD) deficiency).

We asked the pediatricians questions regarding the hour-specific TSB (at 25–48 hours, 49–72 hours and >72 hours) that they used for the initiation of phototherapy and/or exchange transfusion, and the TSB they considered as high risk for the development of kernicterus in term neonates. We also asked about diagnostic approaches (transcutaneous bilirubinometry, cephalocaudal progression), and pre- and post-discharge neonatal hyperbilirubinemia management. Additionally, we sought their opinion on whether severe hyperbilirubinemia and kernicterus should be considered a public health concern and made reportable conditions in the New Jersey.

The questionnaire was pre-tested among seven pediatricians from the university hospital and private practice setting in order to reduce redundancy and increase the clarity of the questions. The Institutional Review Board of the UMDNJ-Robert Wood Johnson Medical School approved the study.

### Statistical analysis

The statistical analysis was performed using STATISTICA 6.0 for Windows (StatSoft, Inc., Tulsa, OK) to identify the significance of the observed differences in proportion (Chi-square test) and the continuous variables (analysis of variance).

We reported results for the overall sample and for groups that were determined by the type of pediatric practice (university hospital, community hospital, private group, and private solo). Age-specific total serum bilirubin (TSB) levels published by the AAP in 1994 for the initiation of phototherapy and/or exchange transfusion were used for assessment of the pediatricians' preference in the treatment of neonatal jaundice [9]. Significant differences were accepted if the P value was less than 0.05 (2-tailed).

## Results

### Response rate and demographic characteristic of the respondents

Among the 431 returned questionnaires, 24 were received incomplete from retired pediatricians, 13 from pediatricians in residency training, and 17 from pediatricians who did not provide neonatal services. Twenty-one questionnaires were returned because of lack of a forwarding address. The rest of the respondents (n = 356) completed more than 84% of the survey questions and were included in the analysis. The adjusted response rate of 49.1% was calculated by dividing the number of completed survey questionnaires (n = 356) by the 725 eligible responders [800-(24+13+17+21)].

The majority of the respondents (90.7%, n = 323/356) were board certified. The demographic and practice characteristics of pediatricians by the type of practice are presented in Table 1. Most of the respondents practiced in private groups and provided services for children from the suburban area of New Jersey. University and community hospital-based practices that most often provided pediatric services for children from urban and rural areas involved only 19.9% of the respondents. Private pediatricians in solo practice were older, had been in practice much longer and provided services for a lesser annual number of neonates.

**Table 1:** Demographic and other characteristics of the pediatricians by the type of practice

Characteristics	Type of Practice					
	
	Total* (n = 356)	University Hospital (n = 29)	Community Hospital (n = 42)	Private Group (n = 219)	Private Solo (n = 66)	P** value
Male	175(49.2%)	18(62.1%)	16(38.1%)	105(47.9%)	36(55.4%)	.108
Age (years) †	45.3 ± 11.5	39.9 ± 12.8	43.3 ± 10.9	45.4 ± 10.8	49.8 ± 12.7	.01
Years after residency*	14.3 ± 1.4	11.3 ± 9.8	12.1 ± 10.1	14.6 ± 9.3	16.5 ± 9.8	.05
Neonates per year††	150	245	300	170	60	.01
Practice area***						
Suburban	302(84.5%)	20(69.0%)	24(57.1%)	203(92.7%)	55(83.3%)	.01
Urban	110(30.9%)	19(65.5%)	27(64.3%)	44(20.1%)	20(30.3%)	.01
Rural	56(15.7%)	5(17.2%)	11(26.2%)	33 (15.1%)	7(10.6%)	.07

* Total number of respondents (University and Community Hospitals, Private Groups and Solo)

** P-values represent Chi-square test (for proportion) and analysis of variance (for continuous variables)

*** Check all that apply

† Mean age ± Standard Deviation (years)

†† Median

### Pediatricians' practice preference for neonatal hyperbilirubinemia management

Overall, a higher proportion of pediatricians checked TSB levels in jaundiced neonates prior to their discharge from the hospital as compared to jaundiced neonates noticed at the infant's post-discharge visit (87.4% vs. 57.7, P < 0.01). Table 2 indicates that pediatricians from the university hospitals showed lower TSB testing activity prior to the jaundiced neonate's discharge from the hospital as compared to pediatricians in private practice. In cases where the mother called for advice regarding the baby's jaundice, the majority of pediatricians asked the mother to bring the infant to the office and only 12% of them preferred to directly refer the infant to a laboratory in order to obtain a TSB level. None of the pediatricians advised the mother to stop breastfeeding. Most respondents used cephalocaudal progression of jaundice and a significantly low number of pediatricians (mainly in solo practice) used transcutaneous bilirubinometry (TcB) for the quantification of neonatal jaundice.

**Table 2:** Pediatricians' preferences regarding the management of neonatal jaundice

	Practice Type					
	
	Total*	University Hospital	Community Hospital	Private Group	Private Solo	P** value
TSB testing with clinical jaundice before discharge	306/350 (87.4%)	16/24 (66.7%)	39/42 (92.9%)	195/218 (89.5%)	55/64 (85.9%)	0.012
TSB testing with clinical jaundice post-discharge	196/340 (57.7%)	12/24 (50.0%)	28/37 (75.7%)	116/214 (54.2%)	39/64 (60.9%)	0.302
Using cephalocaudal assessment†	271/348 (77.9%)	21/28 (75.0%)	34/41 (82.9%)	170/217 (78.3%)	46/62 (74.2%)	0.645
Using TcB assessment†	56/349 (16.1%)	5/27 (18.5%)	10/42 (23.8%)	34/216 (15.7%)	7/64 (10.9%)	0.017
Recommendations to the mother regarding the baby's jaundice						
1. Bring baby to the office	284/335 (84.8%)	20/25 (80%)	35/40 (87.5%)	175/210 (83.3%)	53/60 (88.3%)	0.426
2. Put baby in the sunlight	3/335 (1.1%)	-	1/40 (2.5%)	2/210 (0.95%)	-	
3. Refer baby for TSB measurement	38/335 (11.4%)	3/25 (12.0%	3/40 (7.5%)	26/210 (12.4%)	6/60 (10.0%)	
4. Stop breastfeeding	-	-	-	-	-	
5. Other	9/335 (2.7%)	2/25 (8.0%)	-	7/210 (3.3%)	1/60 (1.7%)	

*Total number of respondents (University and Community Hospitals, Private Groups and Solo)

** P-values represent Chi-square test (for proportion)

†To quantify the severity of jaundice

### Treatment pattern of neonatal hyperbilirubinemia

**Figure 1:**
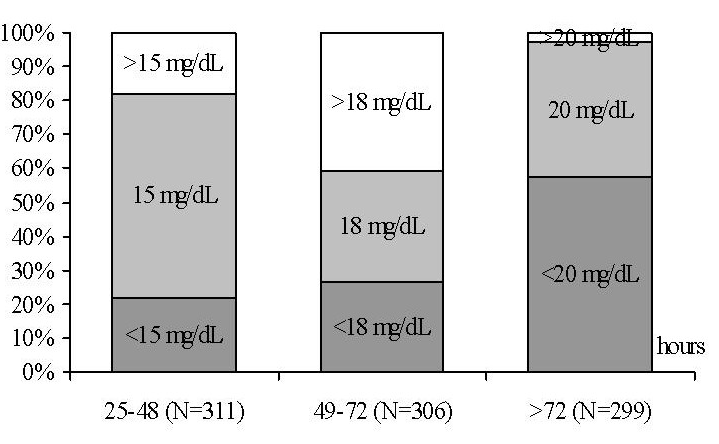
The proportion of pediatricians who practiced phototherapy treatment for term infants in accord with the AAP recommended [9] hour-specific TSB levels (mg/dL) at 25–48 hours (≥15 mg/dL), 49–72 hours (≥18 mg/dL), and >72 hours (≥20 mg/dL).

**Figure 2:**
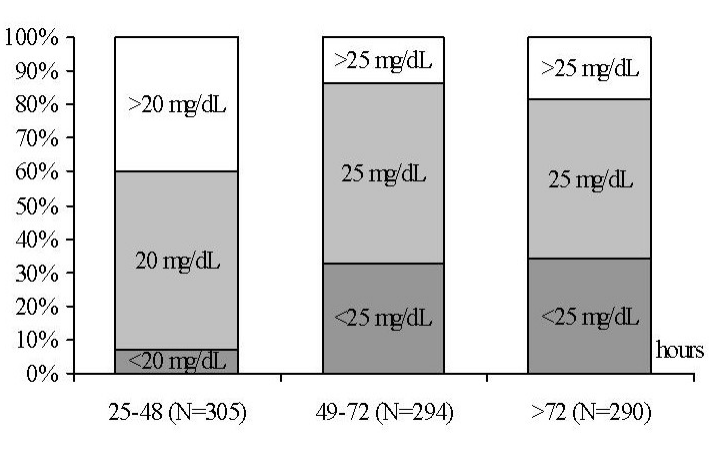
The proportion of pediatricians who practiced exchange transfusion if intensive phototherapy fails for term infants in accord with the AAP recommended [9] hour-specific TSB levels (mg/dL) at 25–48 hours (≥20 mg/dL), 49–72 hours (≥25 mg/dL), and >72 hours (≥25 mg/dL).

Pediatricians' practices regarding phototherapy use in neonates with respect to age-specific TSB levels are revealed in Figure 1. Initiation of phototherapy at TSB levels lower than recommended by the AAP at 24–48 (≥15 mg/dL), 49–72 (≥18 mg/dL), and >72 hours (≥20 mg/dL) were reported by 21.8%, 26.4% and 57.2% of the pediatricians, respectively. As shown in Figure 2, exchange transfusions at TSB levels lower than recommended by AAP at the age of 24–48 (≥20 mg/dL), 49–72 (≥25 mg/dL), and >72 hours (≥25 mg/dL) were reported by 6.7%, 32.7%, and 34.2% of the respondents, respectively. The pediatrician's preference for using age-specific thresholds of TSB for phototherapy and exchange transfusion was not associated with the type of practice.

### Risk factors for hyperbilirubinemia and kernicterus

The pediatricians' response regarding risk factors for the development of severe neonatal hyperbilirubinemia is presented in Table 3. The majority believed "to a very high or moderate degree" that jaundice presenting in the first 24 hours and Rh/ABO incompatibility were significant risk factors for the development of hyperbilirubinemia in term infants. More than 70% of the pediatricians stated that "hardly ever or to a small degree" would they consider gestational age between 37–38 weeks and jaundice noted after discharge from the hospital as significant risk factors for the development of severe neonatal hyperbilirubinemia. Breast-feeding, bruising/cephalohematoma, and previous siblings with jaundice were rated by more than 80% of pediatricians' as risk factors for hyperbilirubinemia with only a small or moderate degree of belief. The pediatricians' belief regarding the relationship between these risk factors and severe hyperbilirubinemia was not associated with the practice type.

**Table 3:** Pediatricians' answers to the question: "Do you believe that following factors are associated with severe hyperbilirubinemia in term neonates?"

Risk factor and number of respondents	Response				
	
	Hardly at all	To a small degree	To a moderate degree	To a very high degree	Not applicable
Jaundice presenting in the first 24 hours (n = 348)	2.3%	3.5%	17.2%	77.0%	-
Jaundice noted at discharge (n = 345)	14.2%	52.5%	29.3%	2.9%	1.2%
Gestational age between 37 and 38 weeks (n = 346)	25.1%	45.7%	26.0%	2.0%	1.2%
Breastfeeding (n = 343)	12.0%	42.0%	40.2%	5.8%	-
Bruising and/or cephalohematoma (n = 345)	2.6%	38.6%	50.4%	8.4%	-
Rh incompatibility (n = 347)	4.0%	6.9%	25.9%	61.9%	1.2%
ABO incompatibility (n = 342)	0.9%	8.8%	46.5%	43.6%	0.3%
G-6-PD deficiency (n = 339)	5.9%	17.4%	40.4%	34.2%	2.1%
Previous sibling with jaundice (n = 346)	15.6%	45.1%	34.4%	4.3%	0.6%

**Figure 3:**
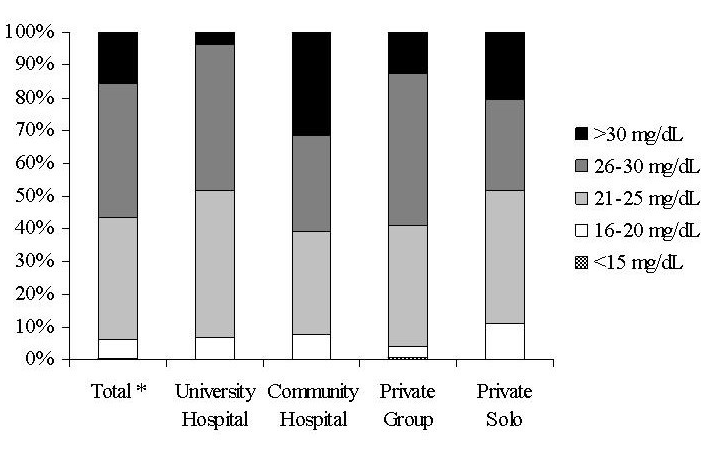
Pediatricians' beliefs regarding TSB levels (mg/dL) at risk for kernicterus* Total number of respondents (University and Community Hospitals, Private Groups and Solo Practice).

Only 48 of the 351 respondents (13.9%) reported first hand experience with one or more patients with kernicterus. These pediatricians were older (50.7+/-11.5 vs. 44.5+/-11.3 years, P < 0.03) and had been in practice much longer (18.0+/-12.4 vs. 13.8 +/-12.4 years, P < 0.01) as compared those who had not seen a single case of kernicterus. A large number of pediatricians believed that a TSB more than 20 mg/dL was a significant risk factor for the development of kernicterus (Figure 3). Among these, a higher proportion of pediatricians from community hospitals regarded bilirubin >30 mg/dL as a risk factor for kernicterus. The vast majority of respondents (more than 88% in each practice group) rated kernicterus as a public health concern and about 50% agreed that severe hyperbilirubinemia and kernicterus should be made laboratory based reportable conditions in New Jersey.

## Discussion

The result of our population-based survey of practicing pediatricians in New Jersey showed overall uniformity with the 1994 AAP recommendations [9] in the management of neonatal hyperbilirubinemia prior to discharge but significant heterogeneity in the post-discharge follow up and treatment practices. Although the majority of respondents preferred to see infants with jaundice in their office, most did not consider post-discharge jaundice as an indicator for a follow up TSB level. Such post-discharge follow up practices may contribute to the development of undiagnosed pathological neonatal hyperbilirubinemia because of the practice of early discharge and the presentation of jaundice mostly after the third day of life [10,11]. However, almost all of the respondents were concerned about bilirubin-induced neurological complications and recognized the importance of post-discharge TSB monitoring. Therefore, the lack of outcome expectancy that is often classified as the major factor influencing physicians' compliance with existing recommendations [12] could not be considered. In addition, approximately 60% of the pediatricians reported the initiation of phototherapy in neonates more than 72 hours old at TSB levels lower than recommended by the AAP [9]. Gartner et al (although the age of the infants was not reported) previously revealed this general tendency for the initiation of phototherapy at lower TSB levels in a pediatrician's survey in 1992 [5]. Concern has been expressed regarding negative outcome in association with the use of lower threshold bilirubin levels for the initiation of therapy for neonates with hyperbilirubinemia [13-15]. The initiation of phototherapy at TSB lower than the AAP recommended levels by some pediatricians may reflect the insufficiency in our understanding of the biology of neonatal jaundice [16].

The majority of respondents reported using cephalocaudal progression of jaundice to quantify the severity despite the inaccuracy of this methodology especially in darkly pigmented infants [2,17]. The low utilization of TcB for assessment of the severity of neonatal hyperbilirubinemia that was reported by the majority of pediatricians may reflect their uncertainty regarding the diagnostic accuracy of this methodology [18-22] or the cost of the equipment. It is possible that the disagreement that exists in the literature [23-29] alters the practicing pediatricians' perception regarding the importance of some significant risk factors in the development of severe hyperbilirubinemia.

Certain aspects of our study such as the response rate of 49.1%, may limit the interpretation of the results. However, this response rate may actually represent a higher proportion of the general pediatricians because the mailing list of the New Jersey Chapter of the AAP included pediatricians who were in specialty practice and the likelihood of a response from these physicians was rather small. Moreover, studies have shown that non-responder bias is not strongly related to the survey response rate and therefore it is unlikely that a greater survey response rate would have significantly altered the results [30,31]. The demographic composition of our respondents is no different from that of the AAP Fellows in the rest of the United States (Table 4). Secondly, we did not review records to confirm actual practices. However, previous studies have shown that surveyed physicians are able to characterize their actual practices with reasonable accuracy [32]. Thirdly, junior fellows in residency programs and family practitioners who may provide neonatal services were not included in the study, but that is a relatively small group.

**Table 4:** Demographic characteristics of the total population of AAP Fellows in the United States versus respondents

Demographic characteristics	AAP data for the U.S. *	Respondents†	P value
Gender			.328
Male	52.3%	49.2%	
Female	47.7%	50.8%	
Age			
<34 years	24.2%	19.4%	.071
35–44 years	34.5%	31.8%	.550
45–54 years	25.3%	30.0%	.100
55–64 years	11.9%	13.5%	.444
>65 years	4.2%	5.3%	.405

* N = 820 AAP fellows (Socioeconomic Survey of Pediatricians: Part 1 2000, Response rate 52%)

† N = 356 respondents

## Conclusion

In conclusion, the apparent low threshold of bilirubin level for the pediatrician's concern regarding kernicterus and their willingness to initiate phototherapy or exchange transfusion at TSB levels well below those recommended by the AAP are the most important results of this study. The pediatricians are aware of the message regarding the importance of preventing severe hyperbilirubinemia and hyperbilirubinemia-related neurological complications as articulated in the AAP guidelines published in 2004 [2]. However, the result of this survey indicates the pediatricians' uncertainties about the utilization of diagnostic approaches and risk factor identification, and their significant tendency for lower utilization of bilirubin levels post-discharge for the initiation of phototherapy. This suggests the need for greater education in order to promote evidence-based practices for the prevention and management of neonatal hyperbilirubinemia and kernicterus.

## Abbreviations

AAP, American Academy of Pediatrics; TSB, serum bilirubin; TcB, transcutaneous bilirubin; G-6-PD, glucose-6-phosphate dehydrogenase.

## Funding/support

**G**rant number MM-0523-03-02/02 from the CDC supported the described project. Its contents are solely the responsibility of the authors and do not necessarily represent the official views of the CDC.

## Pre-publication history

The pre-publication history for this paper can be accessed here:


